# Construction and analysis of circular RNA-associated competing endogenous RNA network in the hippocampus of aged mice for the occurrence of postoperative cognitive dysfunction

**DOI:** 10.3389/fnagi.2023.1098510

**Published:** 2023-03-27

**Authors:** Mingzhu Zhang, Zizheng Suo, Yinyin Qu, Yuxiang Zheng, Wenjie Xu, Bowen Zhang, Qiang Wang, Linxin Wu, Shuai Li, Yaozhong Cheng, Ting Xiao, Hui Zheng, Cheng Ni

**Affiliations:** ^1^Department of Anesthesiology, National Cancer Center/National Clinical Research Center for Cancer/Cancer Hospital, Chinese Academy of Medical Sciences and Peking Union Medical College, Beijing, China; ^2^Department of Anesthesiology, Peking University Third Hospital, Beijing, China; ^3^State Key Laboratory of Molecular Oncology, Department of Etiology and Carcinogenesis, National Cancer Center/National Clinical Research Center for Cancer/Cancer Hospital, Chinese Academy of Medical Sciences and Peking Union Medical College, Beijing, China

**Keywords:** circRNA, ceRNA network, metabolic process, neural plasticity, postoperative cognitive dysfunction

## Abstract

Circular RNAs are highly stable single-stranded circular RNAs and enriched in the brain. Previous studies showed that circRNAs, as part of competing endogenous RNAs (ceRNAs) network, play an important role in neurodegenerative and psychiatric diseases. However, the mechanism of circRNA-related ceRNA networks in postoperative cognitive dysfunction (POCD) has not been elucidated yet. POCD usually occurs in elderly patients and is characterized by hippocampal dysfunction. Here, aged C57BL/6 mice were subjected to exploratory laparotomy under sevoflurane anesthesia, and this POCD model was verified by Morris water maze test. Whole-transcriptome sequencing was performed on the hippocampus of control group (Con) and surgery group. One hundred and seventy-seven DEcircRNAs, 221 DEmiRNAs and 2,052 DEmRNAs were identified between two groups. A ceRNA network was established with 92 DEcircRNAs having binding sites with 76 DEmiRNAs and 549 target DEmRNAs. In functional enrichment analysis, a pathological pattern of POCD was highlighted in the ceRNA network: Abnormal metabolic process in neural cells, including oxygen metabolism, could promote apoptosis and then affect the synaptic function, which may undermine the neural plasticity and eventually lead to changes in cognitive function and other behavioral patterns. In conclusion, this specific ceRNA network of circRNAs–miRNAs–mRNAs has provided novel insights into the regulatory mechanisms of POCD and revealed potential therapeutic gene targets.

## Introduction

Circular RNAs (circRNAs) are one of the noncoding RNAs (ncRNAs), that are highly abundant and evolutionarily conserved (Memczak et al., 2013). Having the feature of tissue-specific, circRNAs are expressed in all tissues, and are particularly abundant in brain. 80% of the efficiently expressed circRNAs in mouse brain were also detected in human brain (Rybak-Wolf et al., 2015), indicating that the expression patterns of circRNAs were highly conserved in the neural system. Twenty percent of protein-coding genes in the brain produce circRNAs, and most of these circRNA-producing genes are expressed only in the brain, suggesting that circRNAs play an essential role in neuro-specific regulation (Hanan et al., 2017). circRNAs exert the regulatory function by forming competing endogenous RNAs (ceRNAs) networks, in which circRNAs combine with miRNA response elements (MREs) and regulate the target mRNAs. Previous studies reported that circRNAs were associated with neurotransmitter function, neuron maturation and synaptic activity (Hanan et al., 2017; Mahmoudi and Cairns, 2019). circRNAs were found to be enriched and accumulated in synapses and other neuronal tissues as the age grew, associated with the occurrence and development of neurodegenerative and psychiatric diseases (Ashwal-Fluss et al., 2014; Westholm et al., 2014; Rybak-Wolf et al., 2015). For example, circRims2, circTulp4, circElf2, circPhf21a and circMyst4 were expressed by genes with a key regulatory role in neuron and brain development (Rybak-Wolf et al., 2015; You et al., 2015). CircHomer1 was associated with neuronal plasticity and synapses structural changes (You et al., 2015). The well-known CDR1as in mammals interacted with miR-7 and miR-671 and was implicated in synaptic transmission and neuropsychiatric disorders (Hansen et al., 2013; Piwecka et al., 2017). Perioperative cognitive dysfunction (POCD) is one of the most common perioperative complications, especially in elderly patients. Recent studies have shown that certain circRNAs were found to be aberrantly expressed in hippocampus, playing a role in POCD through ceRNA network (Wu et al., 2021). A series of circRNAs were found to regulate hub genes such as Rbm47, Sostdc1, Egfr, Prkacb, Unc13c, Tbx20 and St8sia2 through ceRNA networks, participating in Wnt, P53 and NF-κB signaling pathways, associating with the development of POCD (At the Hop 50's Rock 'n' Roll, 1996; Cao et al., 2020; Wu et al., 2021; Wang et al., 2022). Although accumulating evidence explained the regulatory function of circRNAs in the brain, their roles in the perioperative context are still unknown area to be explored. By constructing cricRNA-related ceRNA networks and identifying hub genes, we may understand the ceRNA related mechanism of POCD development and discover the potential therapeutic targets.

Perioperative neurocognitive disorders (PND) include postoperative delirium (POD) and long-term postoperative cognitive dysfunction (POCD), and it is one of the most common perioperative complications. The risk of POCD increases in the elderly patients (> 60 years), appearing to be 25–40% (Evered et al., 2011). POCD is characterized by impaired memory and attention, and could be accompanied by changes in mood, personality, and behavior, lasting from a few days to several years (Fodale et al., 2010). In addition to cognitive dysfunction, other postoperative complications and mortality may be increased in patients with POCD (Fodale et al., 2010; Leslie, 2017). There were numerous studies on the pathologies of POCD, including neuroinflammation, oxidative stress, neuronal damage, blood–brain barrier (BBB) damage and neurotrophic impairment, but the conclusion on the regulatory mechanisms remains unclear (Netto et al., 2018; Yang et al., 2020; Suo et al., 2022). Meanwhile, it has been proved that POCD shared specific common pathways with some neurodegenerative diseases (Alzheimer’s disease, AD, etc.) and psychiatric disorders (Depression, Schizophrenia, etc.; Paterniti et al., 2002; Patron et al., 2013; Evered et al., 2016). For example, the biomarkers of AD, β-amyloid protein and intraneuronal neurofibrillary tangles (tau), have been found to increase after anesthesia and surgery (Xie et al., 2014).

Among these pathologies, ncRNAs (circRNAs and long-noncoding RNAs, lncRNAs), together with miRNAs, play important roles in regulating related genes at the transcriptional level. CircRNA-089763 was related to POCD in elderly patients who underwent non-cardiac surgery (Zhou et al., 2020). We found that lncRNAs Sancr1/2/3 participated in PND process through regulation of metabolism, oxidative stress and mitochondrial function, and aging and apoptosis (Qu et al., 2020). MiR-190a, miRNA-181b-5p and miRNA-146a alleviated POCD by inhibiting hippocampal neuroinflammation (Chen et al., 2019; Liu et al., 2019; Lu et al., 2019). Previous brain studies indicated that circRNAs played a unique role in the regulation of perioperative brain function through ceRNA networks (Li et al., 2021). This study aimed to establish a perioperative circRNA-related ceRNA network in the hippocampus based on RNA sequencing (RNA-seq). Here we analyzed the possible pathological mechanisms and molecular interactions of the occurrence of POCD, including metabolism, immunity and oxidation, neuronal damage, and apoptosis. Based on the construction and analysis of the ceRNA network, the results further revealed the circRNA related perioperative pathologies of the hippocampus, and the mechanisms of the occurrence and development of POCD. The study also analyzed the valuable predictors and potential therapeutic targets for POCD from the perspective of circRNAs.

## Materials and methods

### Animals

C57BL/6 female mice (18-month-old and weighing 25–30 g) were used in this study. The mice were acclimatized for 2 weeks on a standard housing condition (12-h light/12-h dark cycle, 22 ± 2°C, food and water *ad libitum*). Animal experiments were performed following the guide for the care and use of laboratory animals, and the protocol was approved by the local biomedical ethics committee (No. LA2018085).

### Anesthesia and surgical procedures

The aged mice were randomly assigned to control and surgery groups. The surgery group received 2.5% sevoflurane anesthesia with 50% fresh air and 50% O_2_, as the minimum alveolar concentration (MAC) of sevoflurane for mice was 2.4–2.7% (Li et al., 2014). The mice breathed spontaneously, and the concentration of sevoflurane and oxygen concentrations were continuously monitored with an anesthetic monitor (Datex, Tewksbury, MA, United States). After the surgery group’s mice lost consciousness, a modified exploratory laparotomy was performed (Han et al., 2020). A longitudinal midline incision was made with scissors from the glabella to 0.5 cm proximal to the pubic symphysis, through the skin, subcutaneous tissue, abdominal muscles and peritoneum. Approximately 10 cm of the intestine was exposed outside the abdomen for 2 min. The incision was sutured with 5–0 Vicryl thread. The entire procedure was completed in approximately 30 min. During the surgery, the rectal temperature was maintained at 37 ± 0.5°C. It has been reported that there would be no significant alternation of blood pressure and blood gas during this procedure (Ren et al., 2015). The mice recovered in a chamber containing 100% oxygen and stayed for 10 min after waking. Topical application of 0.3% ofloxacin hydrochloride gel was used to reduce postoperative pain. This surgical procedure could induce POCD in aged mice, which was confirmed in our previous study (Suo et al., 2022). The mice in control group received 50% air/50% O_2_ for the same periods without anesthesia or surgery. The hippocampus tissue of the mice was dissected and stored at –80°C at 48 h after the surgery in both control and surgery groups.

### RNA-seq library preparation and sequencing analysis

Library construction was performed according to the Illumina sample preparation for RNA-seq protocol. The mRNA was enriched by magnetic beads with Oligo (dT) after the samples were qualified. When the enrichment was complete, the mRNA was interrupted into short segments with the addition of a fragmentation buffer. Subsequently, double-stranded cDNA was synthesized by reverse transcription using 6-base random primers. The purified double-stranded cDNA was subjected to terminal reparation, single nucleotide A (Adenine) addition and serial sequencing. The fragment size of double-stranded cDNA was selected by an AMpure XP bead (Beckman coulter, Shanghai, China), and the selected double-stranded cDNA was subjected to PCR enrichment to construct a cDNA library. The RNA-seq library for each sample was constructed and sequenced (Compass Biotechnology, Beijing, China) based on the protocols of Illumina HiSeqTM2500/MiSeq^™^ to generate paired-end reads (150 bp in length). The quality of RNA-seq reads from all the brain tissues was checked using FastQC (v0.11.5, Babraham Institute, Cambridge, United Kingdom). Given the heterogeneity of different batches of data, we just used our own sequencing data rather than combined analysis with online database.

### Identification of differentially expressed genes (DEGs)

Differential expression analysis was performed using the HTSeq v0.5.4p3 to identify differentially expressed circRNAs, miRNAs and mRNAs (DEcircRNAs, DEmiRNAs, and DEmRNAs). To better indicate postoperative changes of circRNA, miRNA and mRNA, we just used *p* < 0.05 as the definition of DEG. Read count data were standardized with TMM, and the differences in expression were analyzed by DEGseq (v1.34.0). The overall distribution of the differential genes was shown by scatter plots.

### Construction and function analysis of the ceRNA network

Considering miRanda has a high accuracy in predicting miRNA-target interactions for brain study in mice model, we used miRanda database (Betel et al., 2010) to predict which DEcircRNAs could bind to DEmiRNAs. DEmRNAs targeted by DEmiRNAs were retrieved based on the miRanda (Betel et al., 2010), PITA (Kertesz et al., 2007) and RNAhybrid (Kruger and Rehmsmeier, 2006) databases, and the intersection of the three databases was selected as candidate DEmRNAs. The potential function of these DEmRNAs was analyzed through the Gene Ontology (GO) functional annotation (Huang da et al., 2009) and Kyoto Encyclopedia of Genes and Genomes (KEGG) pathway enrichment analysis (Xie et al., 2011). Cytoscape 3.8.2 was used to construct the circRNA-miRNA network, miRNA-mRNA network, and circRNA-miRNA-mRNA network.

### Quantitative real-time PCR

Quantitative real-time PCR (qPCR) was performed on the CFX96 Real-Time PCR Detection System (Bio-Rad, Hercules, CA, United States). Amplification mixture consisted of PowerUp SYBR Green master mix (ThermoFisher, Wilmington, DE, United States), 10 μM forward and reverse primers (Invitrogen, Carlsbad, CA, United States) and approximately 1.5 μl of cDNA template. Primer sequences were obtained from the literature and checked for their specificity through *in silico* PCR. Amplification was carried out with an initial denaturation step at 95°C for 2 min, followed by 45 cycles of 95°C for 10 s, 55°C for 30 s and 60°C for 30 s, then 65°C for 2 min in 10 μl reaction volume. All reactions were run in duplicate.

### Morris water maze

The Morris water maze (MWM) test (Sunny Instruments Co. Ltd., Beijing, China) was used to assess the spatial learning and memory of mice after surgery. Morris water maze test consisted of a circular tank (120 cm in diameter and 50 cm high) containing water (23 ± 1°C) that is divided into four quadrants and a platform (10 cm in diameter) located 1 cm below the water in the target quadrant. In the place navigation test, the mice were placed in one quadrant facing the wall of the maze and allowed to explore for the hidden platform for 90 s in each trial (four trials per day with an intertrial interval of 5 min). The time to locate the submerged plat form was recorded (defined as the escape latency). If the platform was not found within 90 s, the mice were guided to the platform, where they stayed for 15 s. Mice underwent daily testing in the water maze from day 1 to 5 after surgery. On postoperative day 6, the submerged platform was removed from the water maze and a spatial probe test was performed for 90 s. The swimming speed, escape latency, times of platform crossing, and the time spent in target quadrant were recorded by a video camera.

### Statistical analysis

The statistical calculations were performed with Graphpad Prism 7.0 software. Quantitative data were presented as the mean ± standard deviation (SD). Non-paired double-tailed Student’s *t*-test was used to identify significant differences between the two groups. The *p* value less than 0.05 was considered statistically significant. The correction for multiple testing was applied using the Benjamini–Hochberg method and genes with an FDR < 0.05 were used in downstream pathway enrichment analyses. Enrichment of pathways from KEGG and GO Biological Processes was assessed with Fisher’s exact test, followed by multiple testing correction with the Benjamini–Hochberg method.

## Results

### DEcircRNAs, DEmiRNAs, and DEmRNAs in the hippocampus

**Figure 1:**
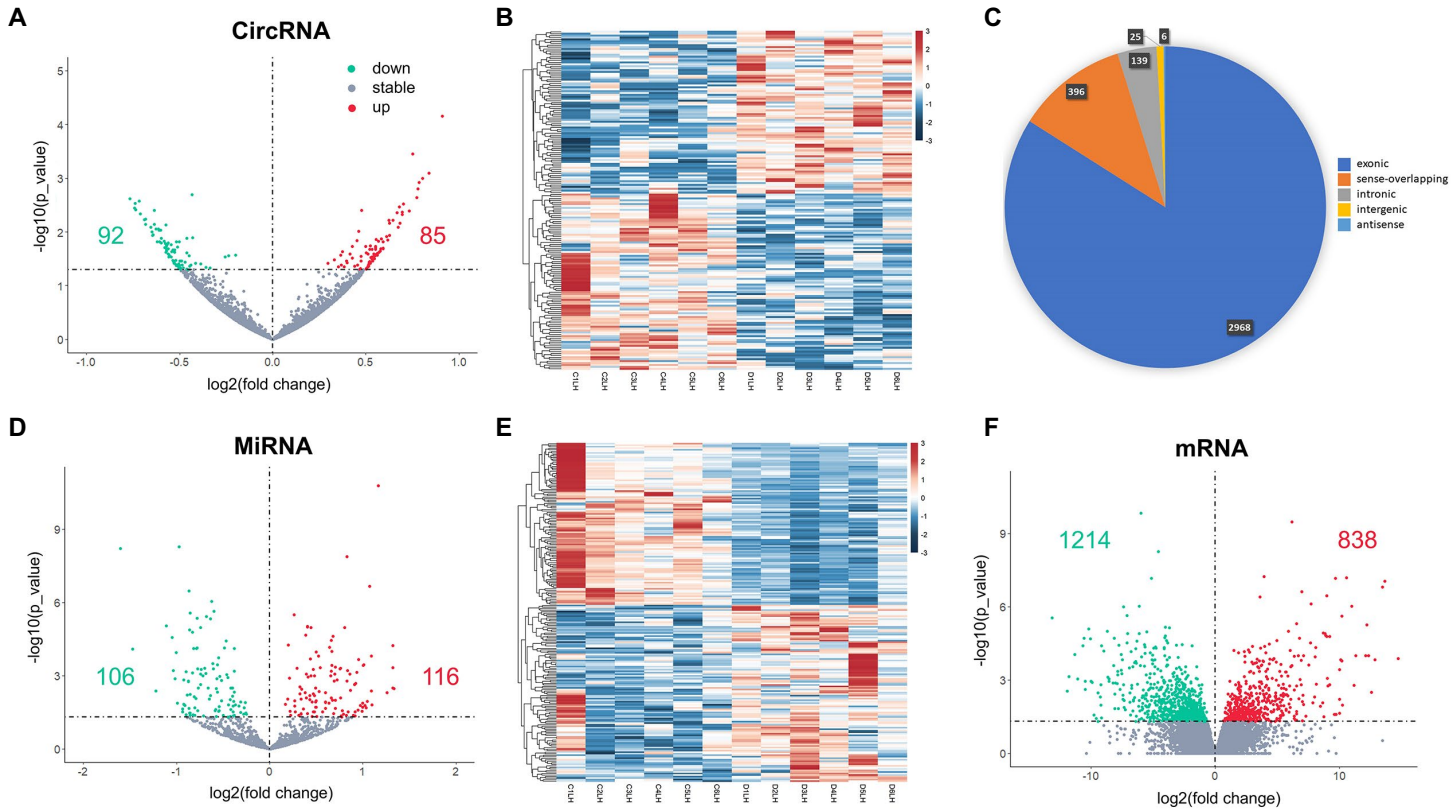
DEcircRNAs **(A,B)**, DEmiRNAs **(D,E)** and DEmRNAs **(F)** in the postoperative period. The classification of DEcircRNAs is shown as pie chart **(C)**. Red dots in volcano plots are up-regulated genes and green ones are down-regulated genes. Red rectangles in heatmaps are up-regulated genes and blue ones are down-regulated genes.

Hippocampus samples from 12 aged mice, including 6 in surgery group and 6 in control group, were enrolled in the study. A total of 3,532 circRNAs were analyzed, and 177 DEcircRNAs (85 up-regulated and 92 down-regulated) were identified between surgery and control groups (*p* < 0.05; Figures 1A,B). A pie chart was made to illustrate the classification of the circRNAs, arranged by their percentage, including exonic (2,966, 84.0%), sense-overlapping (396,11.2%), intronic (139, 3.9%), intergenic (25, 0.7%), and antisense (6, 0.2%) circRNAs (Figure 1C). Exonic circRNAs were the major classification and consisted of certain exonic sequences. These exon-only circRNAs acted as miRNA sponges and were involved in the regulatory functions of target protein-coding genes in cytoplasm. A total of 1,380 miRNAs and 57,825 mRNA were analyzed. Then, 221 DEmiRNA (116 up-regulated and 106 down-regulated; Figures 1D,E), and 2,052 DEmRNA (protein-coding genes, 838 up-regulated and 1,214 down-regulated) were also identified between groups (*p* < 0.05; Figure 1F). We have added the whole lists of DEcircRNAs, DEmiRNAs, and DEmRNAs as Supplementary data 1.

**Figure 2:**
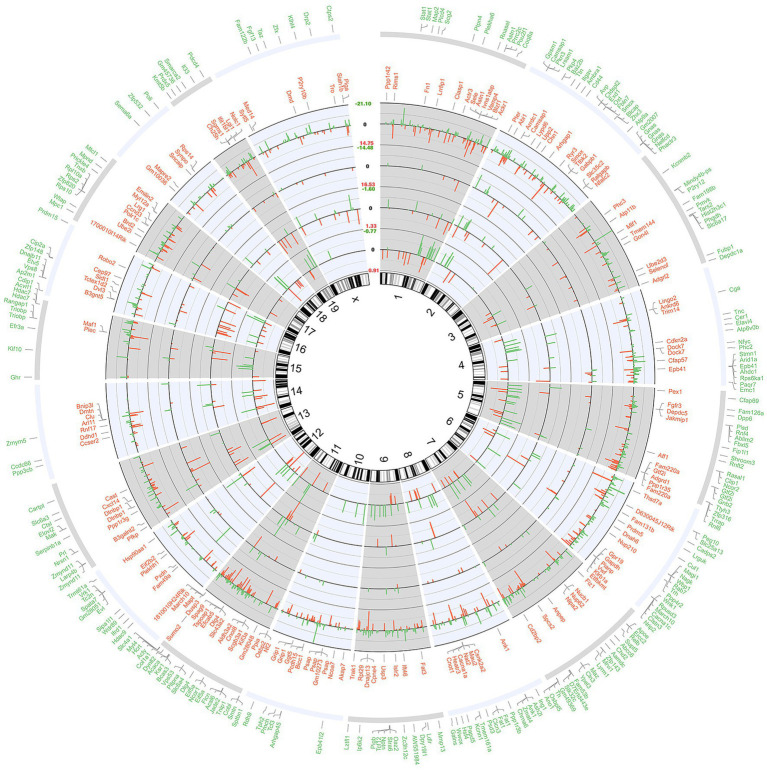
Circular ideogram of the whole transcriptome gene expression. The chromosomal location annotated in a clockwise manner, and the black innermost ring (with vertical lines) represents autosome ideograms (annotated is the chromosomal number), with the pter-qter orientation in a clockwise direction. From the inside to the outside, there were three histograms of gene expression of the 177 DEcircRNAs, 222 DEmiRNAs, and 2,052 DEmRNAs. Red bars inside the ideograms mark genes with increased expression (*p* < 0.05), whereas green bars outside mark those with decreased expression (*p* < 0.05). The length of each bar represented the level of difference (fold change, FC), and the log_2_FC of the RNAs were shown in the blank between chromosome 1 and X. The top 20% protein coding genes (in the descending order of *p* value) that match the Ensemble gene database are listed in the two outermost circles. Red words mean top 20% increased genes (168) and green words mean top 20% decreased genes (243).

Whole transcriptome gene expression in the hippocampus of mice was represented as a circular ideogram composed of concentric circles. From the inside to the outside, five histograms were presented in the order of 177 DEcircRNAs, 221 DEmiRNAs, 2,052 DEmRNA, the top 20% up-regulated and the top 20% down-regulated mRNAs. DEcircRNAs, DEmiRNAs, and DEmRNAs were broadly distributed across 20 mouse chromosomes, either up-regulated (inside red bars) or down-regulated (outside green bars; Figure 2).

**Figure 3:**
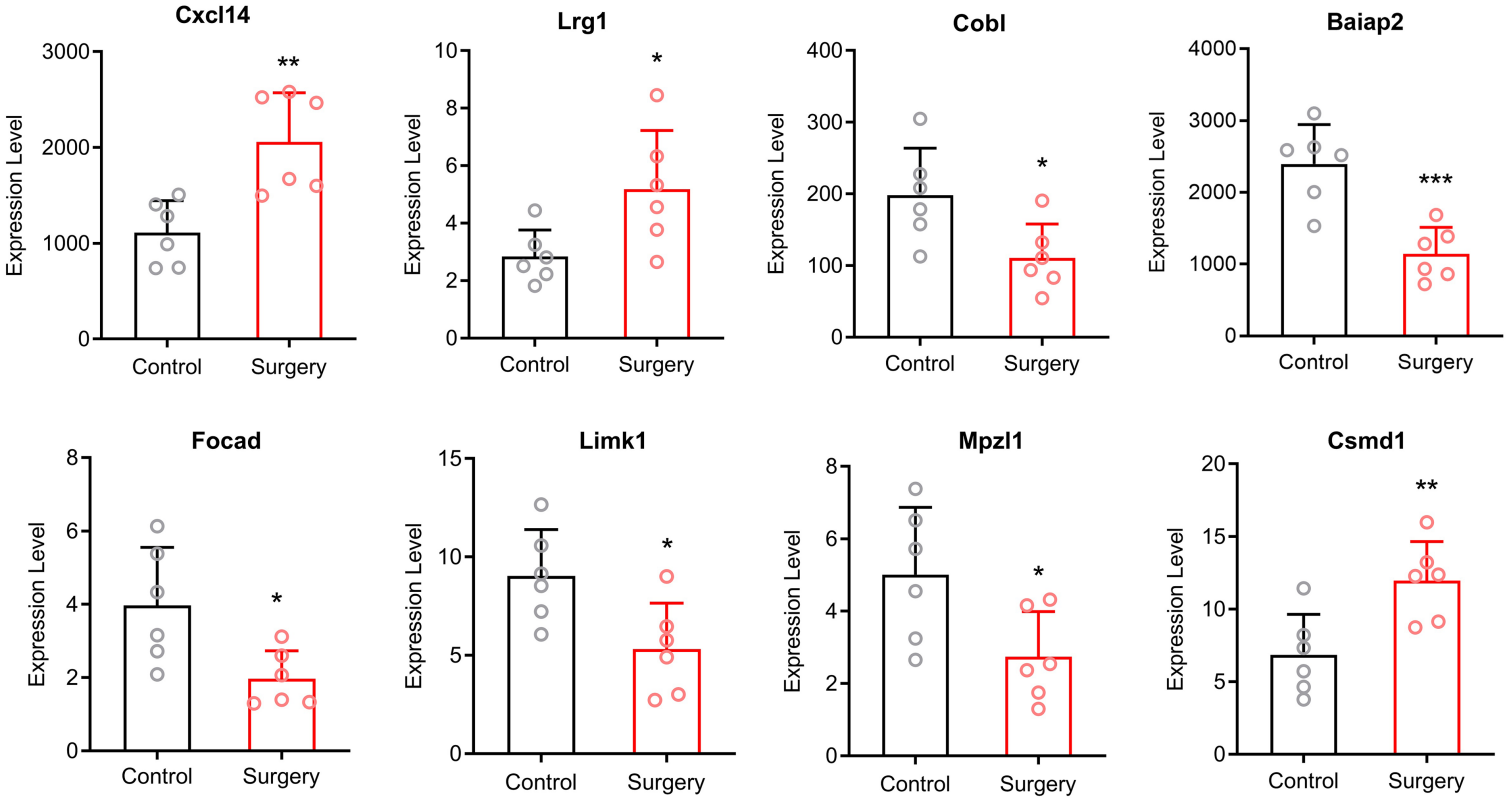
The qRT-PCR validation results. Expression of mRNAs was up-regulated in Cxcl14 and Lrg1, while down-regulated in Cobl and Baiap2 in surgery group compared with control group. Among the circRNAs, Focad, Limk1 and Mpzl1 showed down-regulation, and Csmd1 showed up-regulation in surgery group (Means ± SD, ^*^p < 0.05, ^**^p < 0.01, and ^***^p < 0.001).

Since circRNA competes with mRNA to bind to miRNA, the amount of miRNA is not the primary factor. Therefore, we verified our sequencing results by qRT-PCR of circRNAs and mRNAs. We selected two up-regulated mRNAs and two down-regulated mRNAs with *p* < 0.05, |Log2FC| ≥ 2, as well as four circRNAs with *p* < 0.05. The mRNAs were amplified with convergent primers and the circRNAs were amplified with divergent primers. Expression of mRNAs was up-regulated in ENSMUST00000224801 (Cxcl14) and ENSMUST00000041357 (Lrg1), while down-regulated in ENSMUST00000174874 (Cobl) and ENSMUST00000106233 (Baiap2) in surgery group compared with control group. Among the circRNAs, mmu_circ_0001231 (Focad), mmu_circ_0001427 (Limk1) and mmu_circ_0000114 (Mpzl1) showed down-regulation, and mmu_circ_0001661 (Csmd1) showed up-regulation in surgery group (Figure 3). The validation results were consistent with the normalized expression of RNA-sequence analysis.

### The relationship between DEcircRNA and DEmiRNA, as well as DEmiRNA and DEmRNA

**Figure 4:**
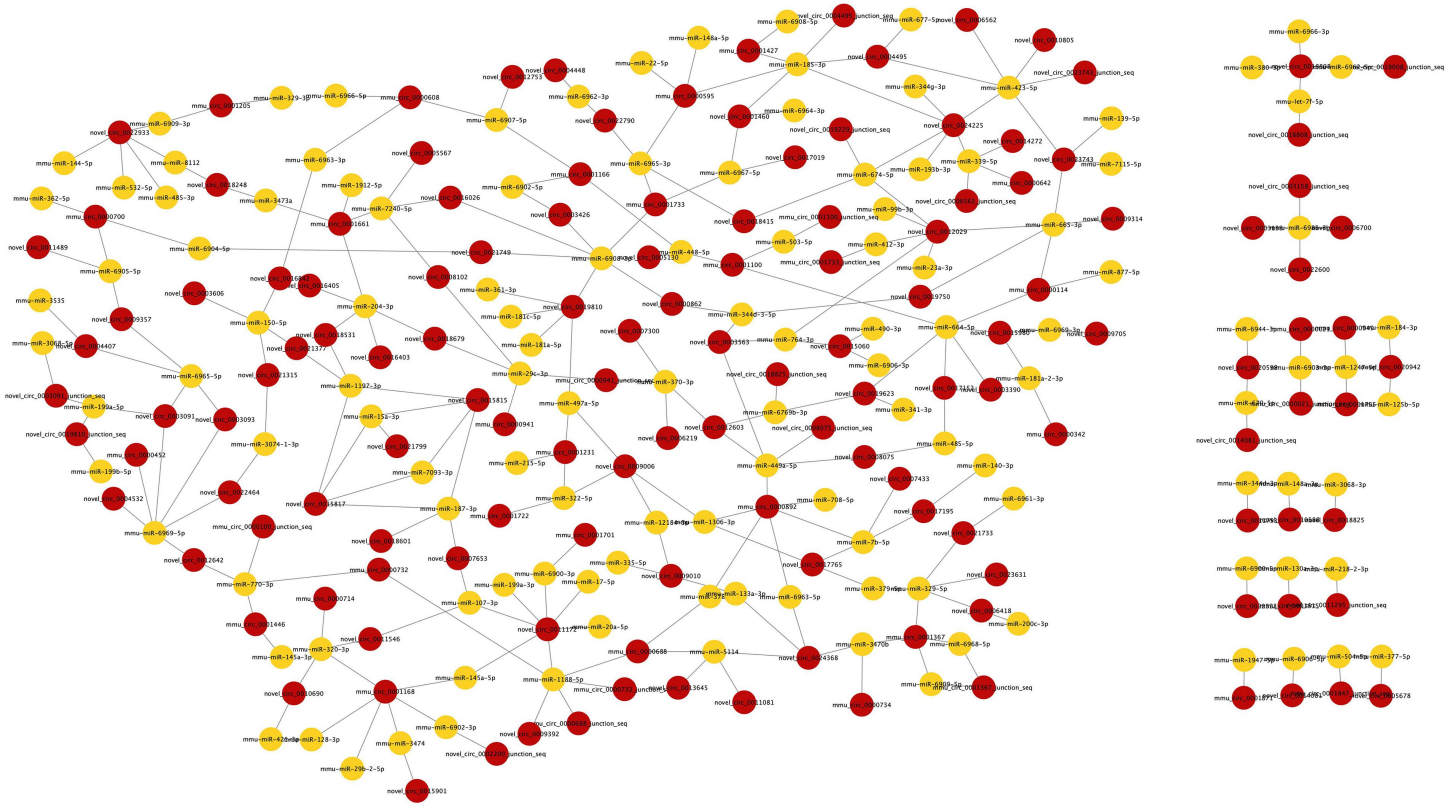
The DEcircRNA–DEmiRNA regulatory network in postoperative period. Circles in red represent DEcircRNAs and circles in yellow represent DEmiRNAs. 98 DEcircRNAs were predicted to bind 95 DEmiRNAs with 190 pairs of interaction.

The miRanda database was used to predict the DEmiRNA-targeted DEcircRNAs. The threshold was set to a maximum binding free energy of −20. Ninety-eight DEcircRNAs were predicted to bind to 95 DEmiRNAs, with 190 pairs of interaction. Cytoscape 3.8.0 was used to build the regulatory network between DEcircRNAs and DEmiRNAs, and the DEcircRNA–DEmiRNA network during surgery was shown in Figure 4.

**Figure 5:**
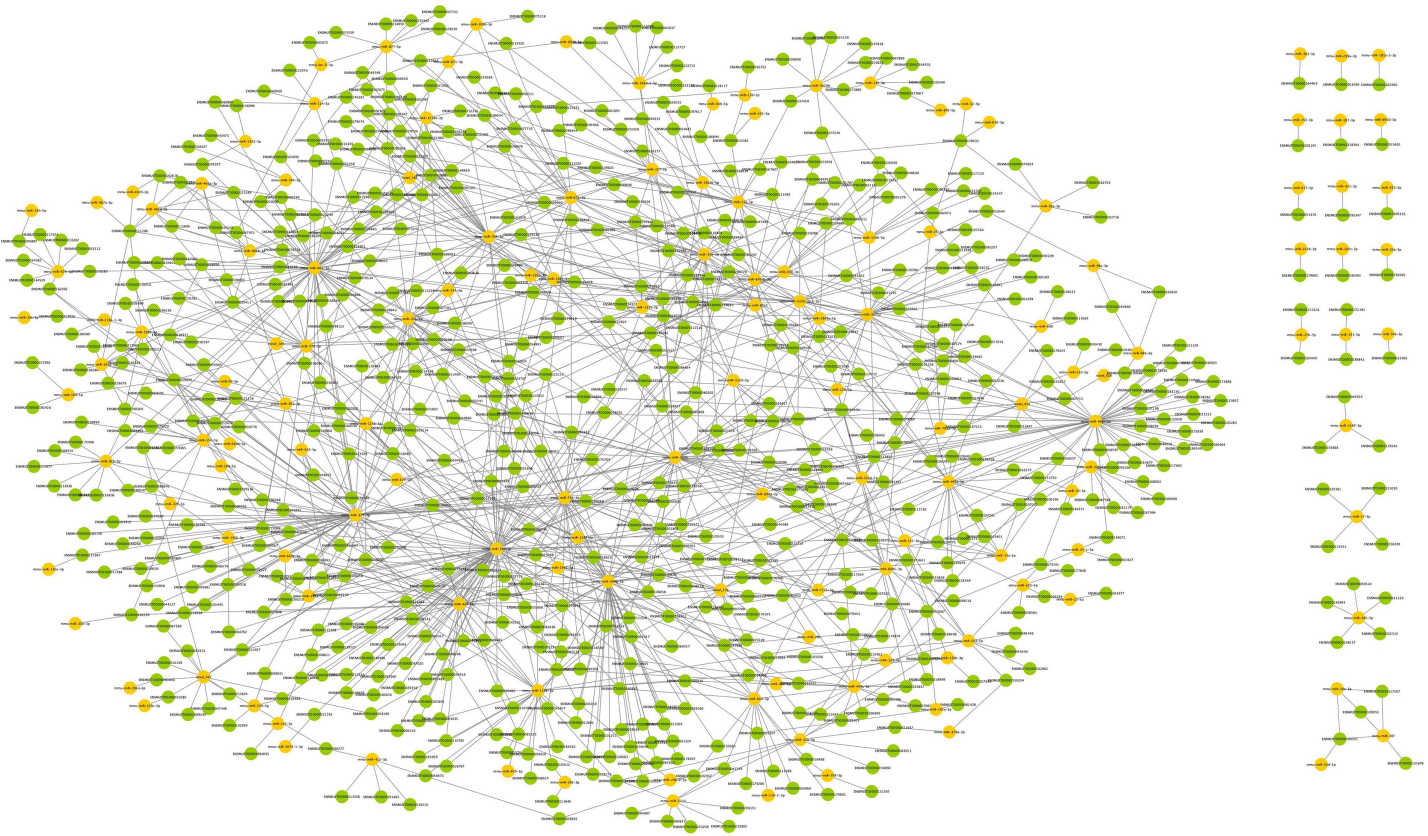
The DEmiRNA–DEmRNA regulatory network in postoperative period. Circles in yellow represent DEmiRNAs, and circles in green represent DEmRNAs. 144 DEmiRNAs were obtained to target 670 DEmRNAs with 1,195 pairs of interaction.

DEmiRNAs were mapped into the miRanda, RNAhybrid and PITA databases to search for their targeting mRNAs. The number of targeted mRNAs in miRanda, RNAhybrid and PITA databases were 18,166, 1,099 and 18,064, respectively. The intersection of these results was further obtained, which included 144 DEmiRNAs, 670 targeted DEmRNAs and 1,195 pairs of interaction. The regulatory network between DEcircRNAs and DEmiRNAs was established using Cytoscape, and the DEmiRNA–DEmRNA network during surgery was shown in Figure 5.

### Functional enrichment analysis and subclassification

The potential biological roles of the 2,052 DEmRNAs were predicted through GO annotation and KEGG pathway enrichment analysis, revealing an enrichment of 1762 GO terms (153 cell components, CC, 256 molecular functions, MF, and 1,353 biological processes, BP) and 15 KEGG pathways (*p* < 0.05). The top terms with repetitive significance were screened and ranked by the number of related genes in the histogram.

**Figure 6:**
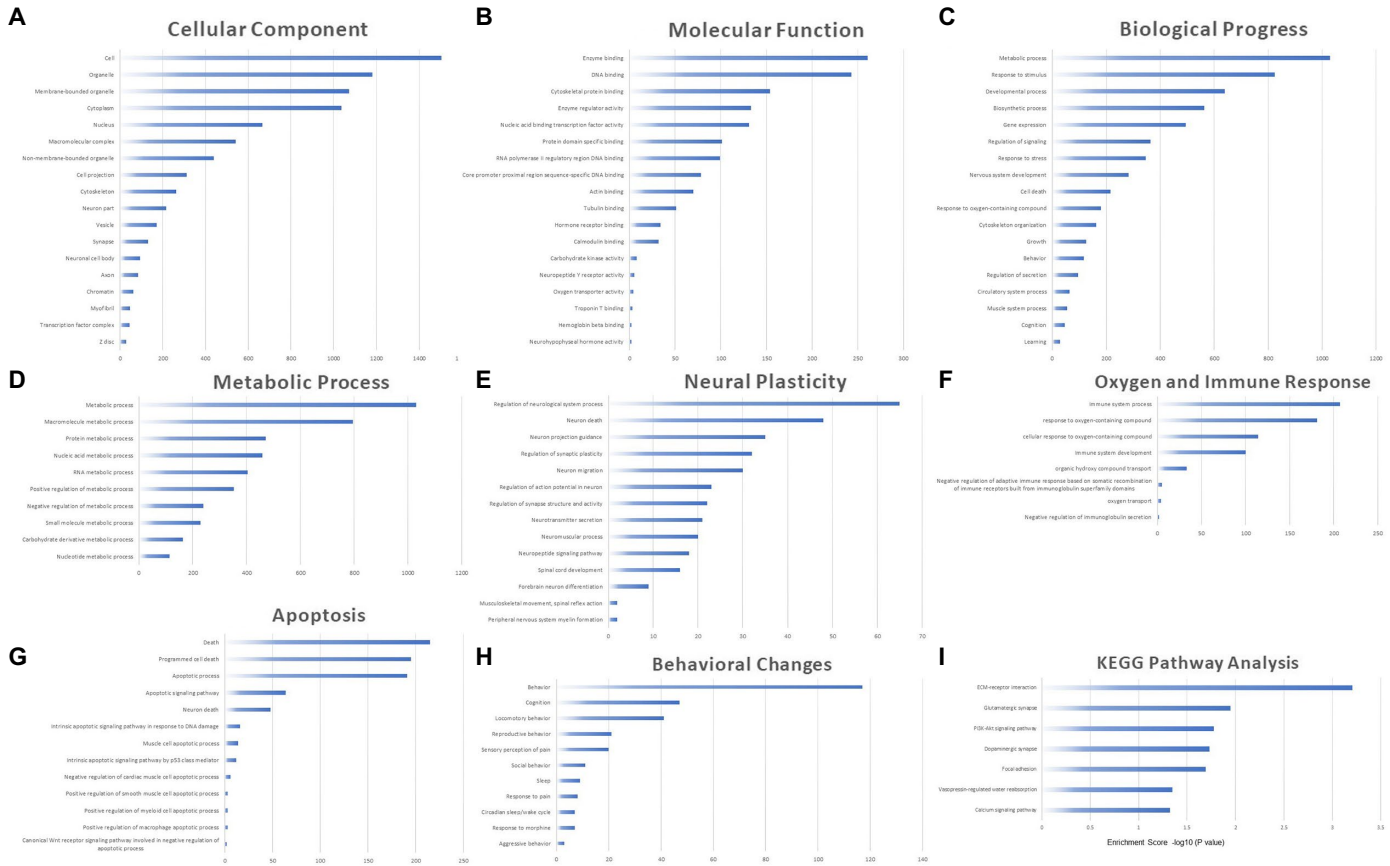
The biological role of 2,052 DEmRNAs through GO annotation and KEGG pathway enrichment analysis. The enrichment of 1762 GO terms including 153 cell components **(A)**, 256 molecular functions **(B)**, 1,353 biological processes **(C)** and 15 KEGG pathways **(I)** (*p* < 0.05). Sub-categories of BP terms including metabolic process **(D)**, neural plasticity **(E)**, apoptosis **(F)**, behavior **(G)** and oxygen and immune response **(H)**.

Cell components terms were mainly enriched in the cell, organelle, membrane-bounded organelle, cytoplasm, nucleus, etc. The enriched biological processes were widely distributed around the cell. Neural system related parts included neuron part, synapse, neuronal cell body and axon (Figure 6A). MF terms were mainly enriched in enzyme binding, DNA binding, cytoskeletal protein binding, enzyme regulator activity, nucleic acid binding transcription factor activity, etc. (Figure 6B), indicating that regulation of transcription and enzyme were the major molecular processes in the aged hippocampus perioperatively. BP terms were mainly enriched in metabolic process, response to stimulus, developmental process, biosynthetic process, gene expression, etc. (Figure 6C). Among them, terms related to metabolic process, neural plasticity, apoptosis, behavioral changes, oxygen and immune response were highly enriched and were the major pathological processes in the perioperative hippocampus.

We furtherly grouped BP terms into the five categories mentioned above. In the category Metabolic Process, the enriched terms included protein, nucleic acid, carbohydrate, macro- and small-molecule metabolic processes, etc. Both positive and negative regulations were involved, which indicated complex metabolic changes in the perioperative period (Figure 6D). Within the category neural plasticity, the enriched terms included regulation of neurological system process, synaptic plasticity, action potential in neuron, synapse structure and activity. Terms including neuron death, projection guidance and migration were also enriched, suggesting that neuronal alterations were the major pathological processes in the perioperative period (Figure 6E). In category oxygen and immune response, the enriched terms included immune system process and development, response to oxygen-containing compound and related processes, and the main regulatory trends of immune process was negative (Figure 6F). In category apoptosis, the enriched terms included death, programmed cell death, DNA damage, apoptotic process and signaling pathways related to neuron, muscle cell, macrophage, etc. These enriched terms indicated that hippocampal cell injury occurred in the perioperative period (Figure 6G). In category Behavior Change, the enriched terms included multiple behaviors, cognition, pain, sleep, response to morphine, etc., indicating the impacts of anesthesia and surgery on postoperative behaviors, cognitive function, pain and circadian rhythm of aged mice.

According to KEGG pathway analysis, the enriched pathways (*p* < 0.05) included ECM-receptor interaction, glutamatergic synapse, PI3K-Akt signaling pathway, dopaminergic synapse, focal adhesion, calcium signaling pathway (Figure 6I). These pathways have also been reported to be involved in neurodevelopmental or neurodegenerative disease processes (Tsui and Isacson, 2011; Brini et al., 2014; Moretto et al., 2018; Akhtar and Sah, 2020). We have added the results of GO/KEGG enrichment analysis as Supplementary data 2.

### ceRNA network of DEcircRNA–DEmiRNA–DEmRNA

**Figure 7:**
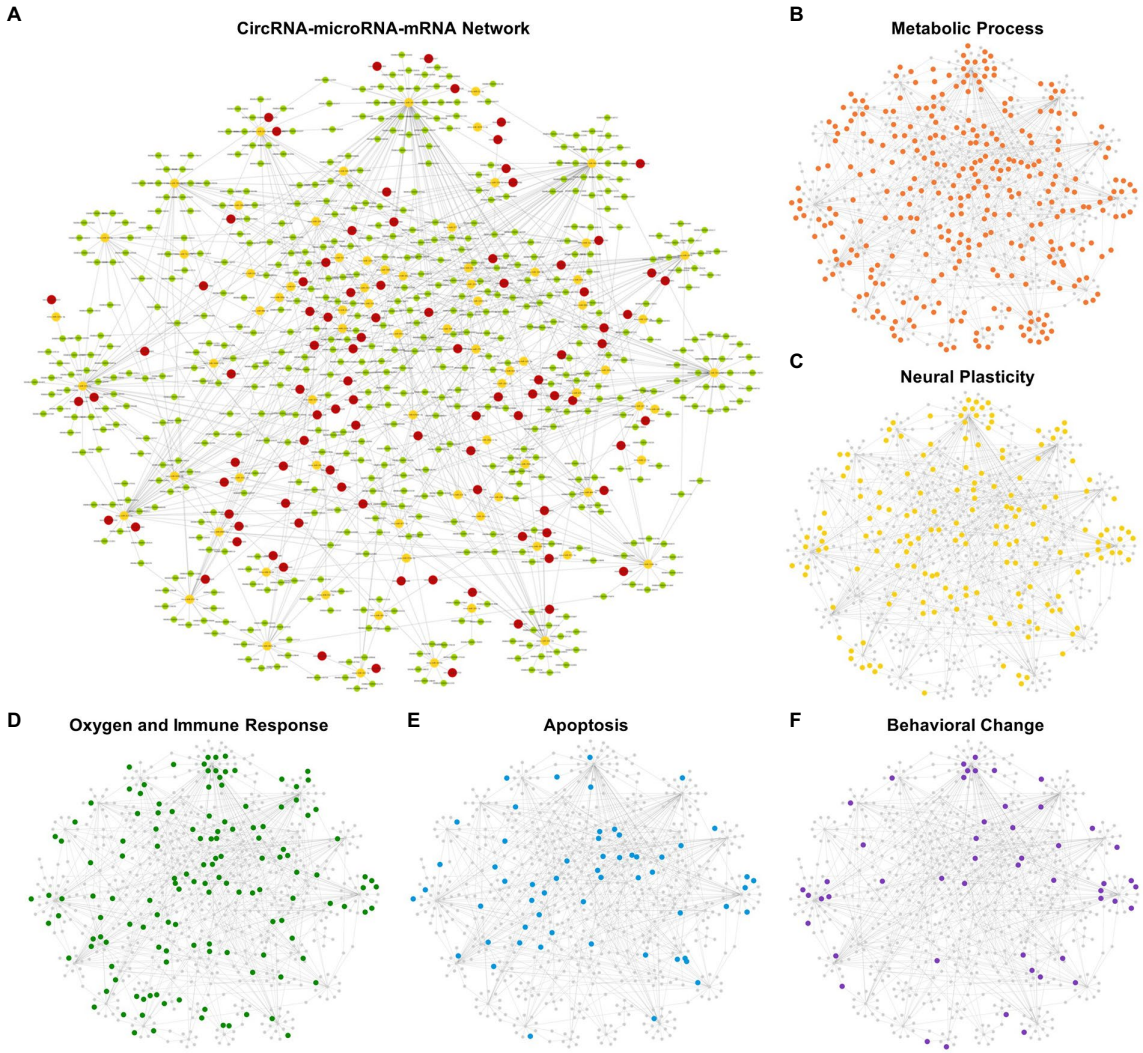
competing endogenous RNA (ceRNA) network of DEcircRNA–DEmiRNA–DEmRNA. 92 DEcircRNAs were obtained to have binding sites on 76 DEmiRNAs with 549 target DEmRNAs. Circles in red represent DEcircRNAs, circles in yellow represent DEmiRNAs and circles in green represent DEmRNAs **(A)**. The mRNAs classified according to the GO analysis were highlighted in the ceRNA network, respectively, as metabolic process, apoptosis, oxygen and immune response, neural plasticity, and behavior related Terms **(B–F)**.

The DEmiRNAs with target DEmRNAs were compared with the DEmiRNAs interacting with DEcircRNAs. Eventually, a ceRNA network was established with 92 DEcircRNAs binding to 76 DEmiRNAs and 549 target DEmRNAs (Figure 7A). We have added the target prediction results of circRNA, miRNA and mRNA as Supplementary data 3. The mRNAs classified according to the GO analysis were highlighted in the ceRNA network as metabolic process, apoptosis, oxygen and immune response, neural plasticity, and behavior related terms, respectively (Figures 7B–F).

Figure 7B shows the ceRNA network related to metabolic processes, in which 310 target mRNAs were involved with 124 up-regulated and 186 down-regulated. The top 5 DEmRNAs (ranked by their variation level between two groups) were *Klf10*, *Hr*, *Coq8a*, *Lrrfip1* and *Kmt5c*. Among them, *Kmt5c* is involved in histone modifications and participates in neural metabolism, neurogenesis and DNA repair. *Kmt5c* was down-regulated in postoperative period. CeRNA network showed *Kmt5c* was negatively regulated by mmu-miR-185-3p, mmu-miR-3,474, and mmu-miR-370-3p. These DEmiRNAs were interacted with novel_circ_0024225, mmu_circ_0001427, mmu_circ_0000595 (mmu-miR-185-3p); novel_circ_0015901 (mmu-miR-3,474); novel_circ_0007300, novel_circ_0006219 (mmu-miR-370-3p). These related DEcircRNAs were all down-regulated like *Kmt5c*. Figure 7C showed neural plasticity related ceRNA network in which 165 target mRNAs were involved with 53 up-regulated and 112 down-regulated. The top 5 DEmRNAs were *Dlg4*, *Map6*, *Hdac7*, *Marveld2* and *Cobl*. Among them, *Dlg4* encodes a major synaptic protein. PSD95 clusters glutamate receptors and is critical for plasticity. *Dlg4* was down-regulated in postoperative period. In the ceRNA network, *Dlg4* was negatively regulated by mmu-miR-665-3p. This DEmiRNA interacted with mmu_circ_0000114, novel_circ_0019750, and novel_circ_0009314. These related DEcircRNAs were all down-regulated like *Dlg4*.

**Figure 8:**
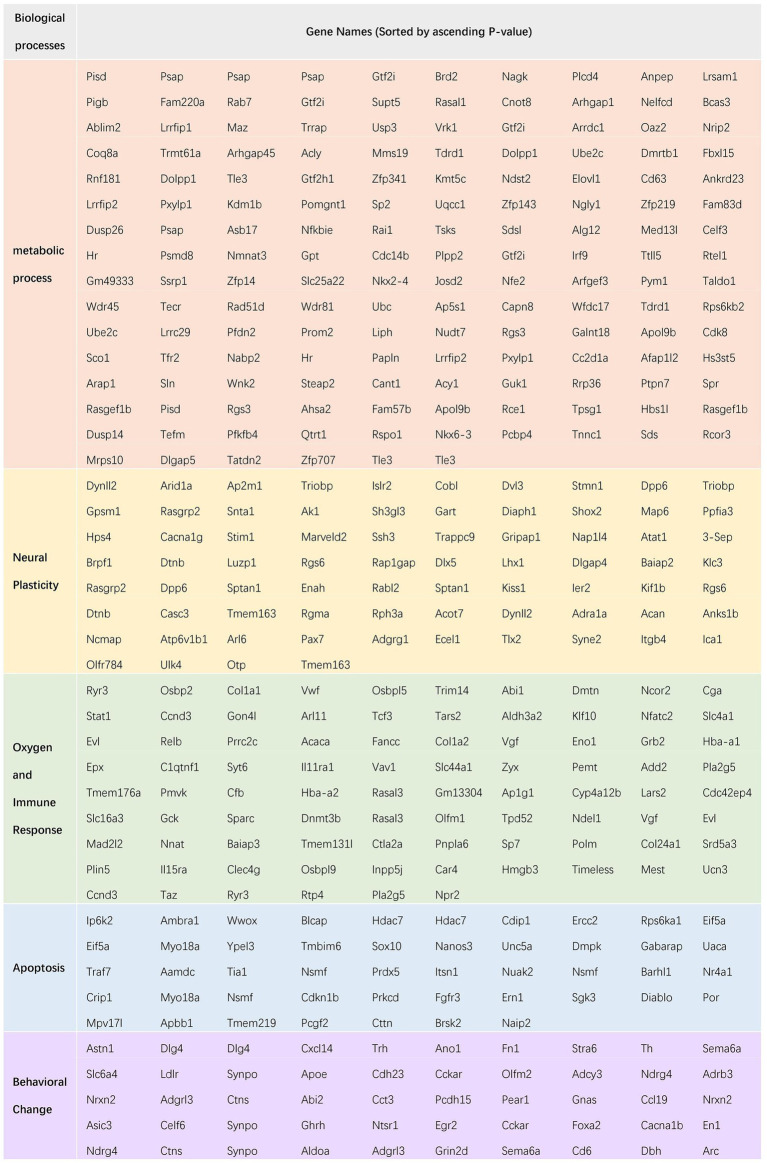
The name of genes encoded by the classified mRNAs in the ceRNA network was shown in the chart, sorted by ascending p value, from left to right.

Figure 7D shows ceRNA network of oxygen and immune response. It included 136 target mRNAs, of which 53 were up-regulated and 83 were down-regulated. The top 5 DEmRNAs were *Ryr3*, *Klf10*, *Dlg4*, *Osbpl5* and *Hdac7*. Among them, *Ryr3* controls the intracellular Ca^2+^ levels and reproduces the core phenotypes of brain aging (i.e., neuroinflammation, neurotoxicity and cognitive deficits) (Liu et al., 2022). *Ryr3* was up-regulated in the postoperative period. The ceRNA network showed *Ryr3* was negatively regulated by mmu-miR-5,114. This DEmiRNA interacted with mmu_circ_0000688, which was up-regulated as *Ryr3* did. Figure 7E showed apoptosis related ceRNA network in which 57 target mRNAs were involved with 24 up-regulated and 33 down-regulated. The top 5 DEmRNAs were *Hdac7*, *Crip1*, *Wwox*, *Nuak2*, *Nsmf*. Among them, *Hdac7* plays an important role in neuroprotection by inhibiting neuron death (Wu and Li, 2022). *Hdac7* was down-regulated in postoperative period. The ceRNA network showed *Hdac7* was negatively regulated by mmu-miR-133a-3p and mmu-miR-674-5p. These DEmiRNAs were interacted with novel_circ_0024368 (mmu-miR-133a-3p); novel_circ_0018415, novel_circ_0024225 (mmu-miR-674-5p). These related DEcircRNAs were all down-regulated as *Hdac7* did. Figure 7F showed behavioral change related ceRNA network in which 50 target mRNAs were involved with 17 up-regulated and 33 down-regulated. The top 5 DEmRNAs were *Dlg4*, *Astn1*, *Ano1*, *Olfm2* and *Synpo*. Among them, *Ano1* encodes a member of Ca^2+^ activated Cl^−^ channels (CaCCs) that are critical for synaptic potential and neuronal signaling, which affects learning and memory processes (Huang et al., 2012). *Ano1* was down-regulated in the postoperative period. The ceRNA network showed *Ano1* was negatively regulated by mmu-miR-370-3p. This DEmiRNA interacted with novel_circ_0007300 and novel_circ_0006219. These related DEcircRNAs were all down-regulated as *Ano1* did. A chart of all the gene name of the five subcategories of biological processes has been showed in Figure 8. From left to right, name of the genes was sorted by ascending *p* value.

### Cognitive function assessed by Morris water maze test

**Figure 9:**
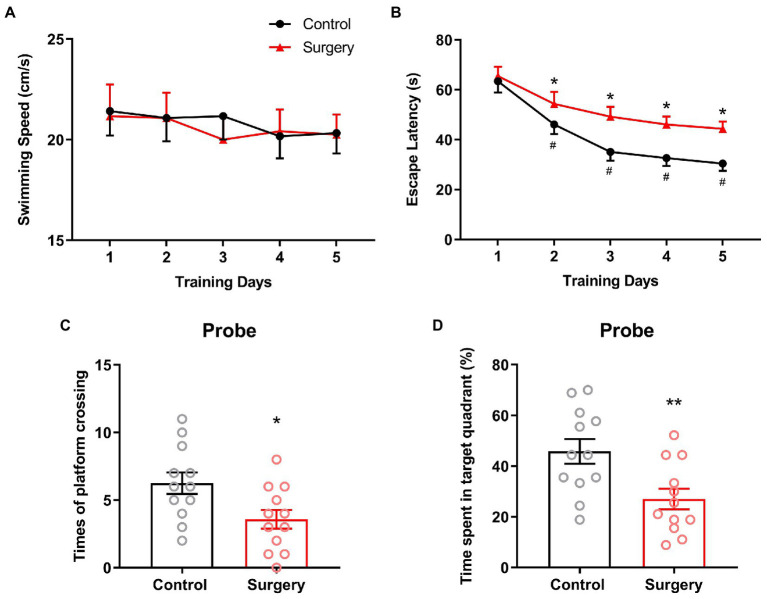
Morris water maze test showed that in the place navigation test, the swimming speed did not change significantly in both groups **(A)**, but the escape latency increased significantly after surgery **(B)**. In the probe test, both the times of platform crossing **(C)** and the time spent in target quadrant **(D)** decreased significantly after surgery. (Means ± SD, ^*^p < 0.05, ^**^p < 0.01).

Twenty-four mice were subjected to Morris water maze test and divided into control and surgery groups (*n* = 12). The place navigation test began at 1 day after surgery, and during five training days, the swimming speed maintained constant and showed no significance between two groups, indicating the intact locomotor activity after surgery (Figure 9A), the escape latency decreased significantly as the training went on and this trend appeared less pronounced in surgery group (Figure 9B). In the probe test (1 day after last training), the times of platform crossing and the time spent in target quadrant decreased significantly in surgery group (6.25 ± 2.73 vs. 3.58 ± 2.39, *p* = 0.0185, 45.83 ± 16.90 vs. 27.04 ± 14.06, *p* = 0.0072; Figures 9C,D). These results indicated the occurrence of cognitive dysfunction and the successful establishment of the POCD model in surgery group, therefore, circRNA associated ceRNA network was involved in the regulation of perioperative cognition.

## Discussion

**Figure 10:**
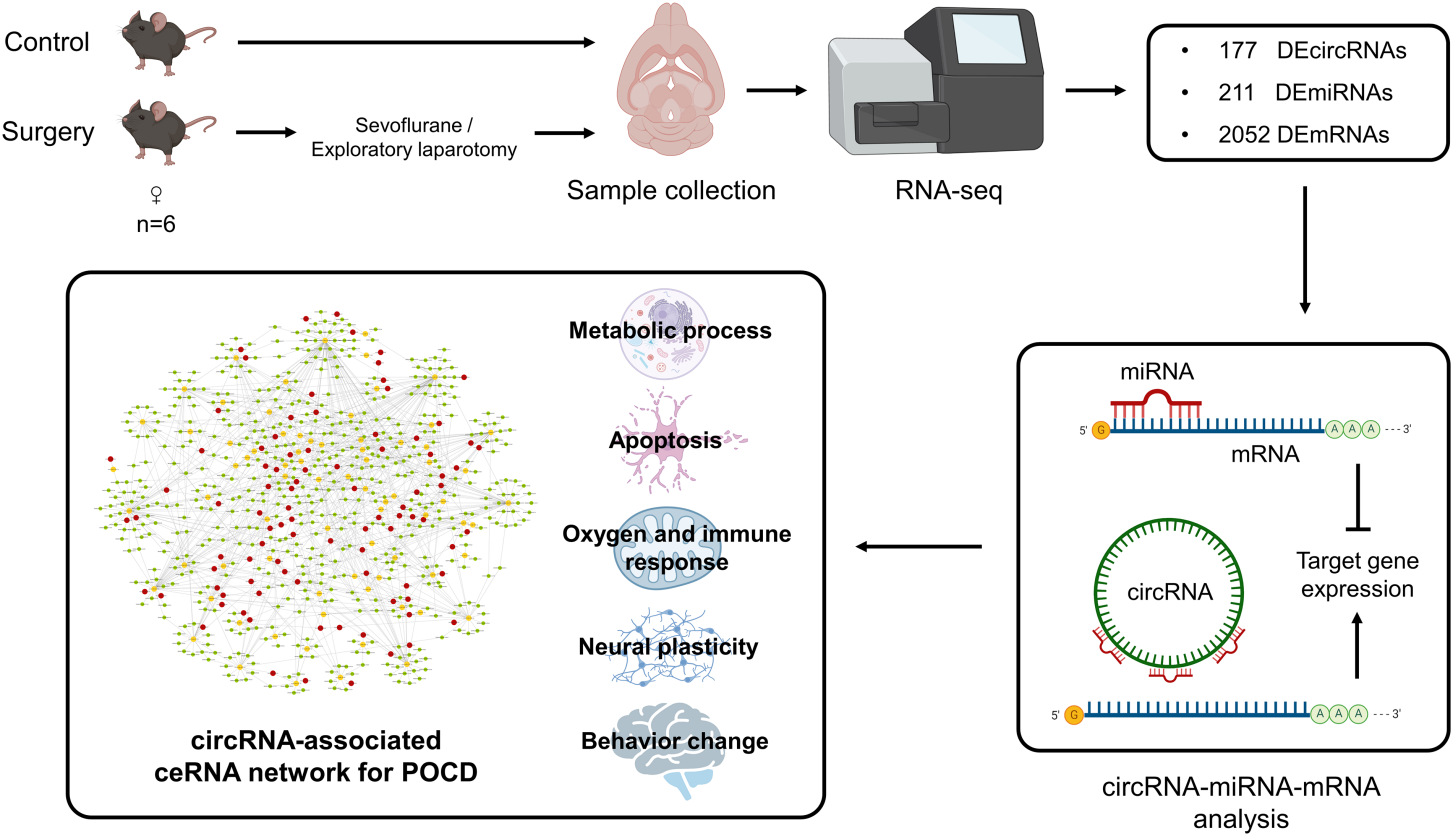
Flow chart of construction and analysis of circRNA-associated ceRNA network for the occurrence of postoperative cognitive dysfunction.

This study focused on the role and impact of circRNAs and related ceRNA network in the pathologies and occurrence of POCD. Previous studies have shown that the aged mice exhibit hippocampus-dependent cognitive dysfunction after exploratory laparotomy and inhaled anesthesia (Qiu et al., 2020; Suo et al., 2022), the present results also indicated the occurrence of POCD in surgery group. The results indicated a total of 177 DEcircRNAs, 221 DEmiRNAs, and 2,052 DEmRNA distributed across 20 chromosomes in the hippocampus of mice after surgery (exploratory laparotomy). The relationship analysis indicated that 98 DEcircRNAs had 95 targeted DEmiRNAs with 190 pairs of interactions, and 144 DEmiRNAs had 670 targeted DEmRNAs with 1,195 pairs of interactions. The enrichment analysis of DEmRNAs revealed 1,762 GO terms and 15 KEGG pathways. The BP terms were further grouped into five functional categories: Metabolic Process, Neural Plasticity, Oxygen and Immune Response, Apoptosis, and Behavior Change. A ceRNA network of DEcircRNA–DEmiRNA–DEmRNA was established, including 92 DEcircRNAs, 76 DEmiRNAs and 549 DEmRNAs, with 1,015 pairs of interaction. Combined with the enrichment analysis, the ceRNA network were further analyzed and presented in a pathological pattern with five functional categories (Figure 10). These results indicated the role of the circRNA-related ceRNA network in the pathogenesis of POCD.

Circular RNAs are highly stable single-stranded circular RNAs that are enriched in the brain. Since circRNAs act as miRNA sponges, it has been speculated that they can transfer signals between cells and are assumed as memory molecules (Lasda and Parker, 2014). Previous studies have revealed the potential regulatory roles of circRNA related ceRNA network in AD (Zhang et al., 2021). Other studies have shown the protective effect of ceRNA network in Parkinson’s disease (Jia et al., 2020) and Stroke (Chen et al., 2021). Several studies have shown that ceRNA network play an important role in numerous physiological and pathophysiological processes, such as neuroplasticity, learning and memory (Zajaczkowski and Bredy, 2021). Many neuron-related circRNAs are associated with synaptic function and differentially expressed during neuronal differentiation and maturation (Rybak-Wolf et al., 2015; You et al., 2015). The expression of circRNAs was throughout the whole life and was dynamically regulated according to different neural activities (You et al., 2015). The increased expression and accumulation of circRNAs was age-dependent, that was related with cognitive deficits in aged animals, but the mechanisms remain unclear (Knupp and Miura, 2018). The dysregulation of circRNAs was involved in the onset and development of neurodegenerative diseases such as Alzheimer’s disease, schizophrenia, autism, depression and other mental diseases (Li et al., 2011; Lukiw, 2013; Liu et al., 2020; Mehta et al., 2020; Zimmerman et al., 2020).

Anesthetics such as sevoflurane acted on neural system, causing learning and memory impairment (Wei et al., 2021). Surgery-induced hippocampal dysfunction led to POCD and long-term memory impairment after POCD occurred (Hovens et al., 2016). Numerous pathological changes in the hippocampus are involved in this process. Neuroinflammation and microglia are the key features. In these cells, NF-κB is activated *via* toll-like receptors and promotes the production of inflammatory cytokines including IL-1β, IL-6 and TNF-α (Yang et al., 2020). Mitochondrial fission and fusion dynamics are also disturbed in the postoperative period (Lu et al., 2020), which leads to a decrease in mitochondrial transmembrane potential and ATP production, which then exacerbates the oxidative stress level. Oxidative stress led to membrane permeability increase, cytochrome c release and respiratory inhibition in hippocampal cells (Starkov et al., 2004). The aforementioned changes also affect synaptic plasticity through dendritic remodeling, influencing on the information flow between neurons. The disruption of tight junction and basement membrane is the mechanism for surgery-induced BBB damage. BBB damage allows the entry of neurotoxic debris, cells and pathogens, which is critical for CNS inflammation and immune responses (Yang et al., 2020). Previous studies have shown that the ceRNA network participated in the above pathological alterations. It plays roles in neuron apoptosis and in regulating multiple signaling pathways, which can lead to hippocampal dysfunction and cognitive disorders (Yu et al., 2021; Zhang et al., 2021). This study provided post-transcriptional evidence for the regulatory potential of circRNA-related ceRNA network for POCD.

In this study, surgery-induced circRNAs were differentially expressed in aged hippocampus. They act as miRNA sponges and regulate the expression of POCD-related genes. These genes were involved in metabolic processes, neural plasticity, oxygen/immune response, apoptosis and behavioral change in postoperative period. Here, novel_circ_0015901 was involved in the regulation of cellular energy expenditure, neurogenesis and DNA repair *via* inhibiting *Kmt5c* expression, which may be associated with longer lasting postoperative cognitive spatial memory impairment (Schotta et al., 2008; Zimmermann and de Lange, 2014). Novel_circ_0009314 was involved in the disruption of synaptic structure and neuronal apoptosis *via* regulating *Dlg4* (known as PSD95; Rubino et al., 2009; Sultana et al., 2010). This suggests that ceRNAs may be involved in the mechanisms of POCD development by affecting neural plasticity as one of the possible ways. Mmu_circ_0000688 was involved in intracellular Ca^2+^ dyshomeostasis *via* promoting the expression of *Ryr3* (Abu-Omar et al., 2018), which may lead to neuroinflammation, impaired LTP, synapse loss and cognition (Futatsugi et al., 1999; Riascos et al., 2011). It also shows that ceRNAs can significantly increase the neurotoxicity of amyloid-β oligomers in aged hippocampal neurons in the above way (Calvo-Rodríguez et al., 2016). Novel_circ_0018415 was involved in postoperative neural apoptosis by regulating the expression of *Hdac7*, which was associated with the activation of c-jun (Evered et al., 2011). Novel_circ_0006219 was involved in postoperative impairment of BBB and cognition, through interaction with mmu-miR-370-3p and suppression of *Ano1*. CaCCs can be affected in this ceRNA network, which may shorten action potential duration, dampen excitatory synaptic potentials, impede temporal summation, and raise the threshold for action potential generation by synaptic potential (Huang et al., 2012). Previous studies also reveal that circRNAs are involved in POCD pathogenesis by modulating the Wnt, VEGF, PKC, neural cell apoptosis and glycolipid metabolism signaling pathways, as well as neural processes associated with long-term synaptic depression and synaptic transmission (Zhang et al., 2022).

This study still has some limitations. First, the surgery group only selected one postoperative time point. Since various postoperative pathological processes are in dynamic change, multiple time points can be set in future studies (such as 2, 7 or 30 days after surgery). Second, this study just provided ceRNA network and the direction for circRNA related pathological processes. For specific pathway or therapeutic targets, intervention studies are still necessary in the future. Third, there are multiple perioperative factors for the occurrence of POCD including anesthesia, surgical trauma, and baseline status of the brain, this study did not analyze their influence, and the conclusion was relatively limited.

## Conclusion

This study identified DEcircRNAs, DEmiRNAs, and DEmRNAs in the hippocampus of aged mice after surgical intervention and sevoflurane anesthesia, and indicated that circRNAs could take part in the occurrence and development of POCD through ceRNA network and protein-coding gene regulation, which are involved in a pathological pattern including metabolic process, neural plasticity and apoptosis. These results provide novel insights into circRNA-related regulatory mechanisms for POCD, and reveal the potential therapeutic gene targets.

## Data availability statement

The results of differential expression analysis (Supplementary data 1), gene enrichment analysis (Supplementary data 2) and target prediction (Supplementary data 3) generated and presented in this study are deposited in the Dryad Digital Repository and will be available here: (https://doi.org/10.5061/dryad.z612jm6gn). The temporary sharing link to download can be found here: https://datadryad.org/stash/share/XxL_uy1jIqeuap9KVWGUjBqHZ3lxdGvPs2EBNy3aPYI.

## Ethics statement

The animal study was reviewed and approved by Biomedical ethics committee of Cancer Hospital, Chinese Academy of Medical Sciences and Peking Union Medical College (No. LA2018085).

## Author contributions

MZ performed the experiments, analyzed the data, and wrote the manuscript. ZS contributed to experiments, data analysis and manuscript revision. YQ contributed to data analysis. YZ and WX contributed to data analysis. BZ, QW, and LW contributed to data analysis and manuscript revision. SL and YC contributed to the manuscript revision. TX contributed to the project supervision and manuscript revision. CN and HZ designed the project, supervised the experiments, drafted and revised the manuscript. All authors contributed to the article and approved the submitted version.

## Funding

This work was supported by the National Natural Science Foundation of China (Nos. 82171195, 81970994, 81771146, and 82101281), Talent Project of National Cancer Center/Cancer Hospital Chinese Academy of Medical Sciences for CN, and Beijing Hope Run Special Fund of Cancer Foundation of China (No. LC2020A01).

## Conflict of interest

The authors declare that the research was conducted in the absence of any commercial or financial relationships that could be construed as a potential conflict of interest.

## Publisher’s note

All claims expressed in this article are solely those of the authors and do not necessarily represent those of their affiliated organizations, or those of the publisher, the editors and the reviewers. Any product that may be evaluated in this article, or claim that may be made by its manufacturer, is not guaranteed or endorsed by the publisher.
